# A system capable of verifiably and privately screening global DNA synthesis

**DOI:** 10.1093/nsr/nwag103

**Published:** 2026-02-16

**Authors:** Carsten Baum, Jens Berlips, Walther Chen, Helena Cozzarini, Hongrui Cui, Ivan Damgård, Jiangbin Dong, Kevin M Esvelt, Leonard Foner, Mingyu Gao, Dana Gretton, Martin Kysel, Juanru Li, Xiang Li, Omer Paneth, Ronald L Rivest, Francesca Sage-Ling, Adi Shamir, Yue Shen, Meicen Sun, Vinod Vaikuntanathan, Lynn Van Hauwe, Theia Vogel, Benjamin Weinstein-Raun, Yun Wang, Daniel Wichs, Stephen Wooster, Andrew C Yao, Yu Yu, Haoling Zhang, Kaiyi Zhang

**Affiliations:** Department of Computer Science, Aarhus University, Aarhus Centrum 8000, Denmark; DTU Compute, Technical University of Denmark, Kongens Lyngby 2800, Denmark; SecureDNA Foundation, Basel 4010, Switzerland; SecureDNA Foundation, Basel 4010, Switzerland; SecureDNA Foundation, Basel 4010, Switzerland; Department of Computer Science and Engineering, Shanghai Jiao Tong University, Shanghai 200240, China; Department of Computer Science, Aarhus University, Aarhus Centrum 8000, Denmark; Institute for Interdisciplinary Information Sciences, Tsinghua University, Beijing 100084, China; SecureDNA Foundation, Basel 4010, Switzerland; Media Lab, Massachusetts Institute of Technology, Cambridge, MA 02142, USA; SecureDNA Foundation, Basel 4010, Switzerland; Institute for Interdisciplinary Information Sciences, Tsinghua University, Beijing 100084, China; Shanghai Qi Zhi Institute, Shanghai 200232, China; SecureDNA Foundation, Basel 4010, Switzerland; Media Lab, Massachusetts Institute of Technology, Cambridge, MA 02142, USA; SecureDNA Foundation, Basel 4010, Switzerland; Department of Computer Science and Engineering, Shanghai Jiao Tong University, Shanghai 200240, China; Institute for Interdisciplinary Information Sciences, Tsinghua University, Beijing 100084, China; Computer Science and Artificial Intelligence Laboratory, Massachusetts Institute of Technology, Cambridge, MA 02139, USA; Computer Science and Artificial Intelligence Laboratory, Massachusetts Institute of Technology, Cambridge, MA 02139, USA; SecureDNA Foundation, Basel 4010, Switzerland; Department of Applied Mathematics, Weizmann Institute of Science, Rehovot 76100, Israel; BGI Research, Changzhou 213299, China; Department of Political Science, Massachusetts Institute of Technology, Cambridge, MA 02142, USA; Computer Science and Artificial Intelligence Laboratory, Massachusetts Institute of Technology, Cambridge, MA 02139, USA; SecureDNA Foundation, Basel 4010, Switzerland; SecureDNA Foundation, Basel 4010, Switzerland; SecureDNA Foundation, Basel 4010, Switzerland; BGI Research, Changzhou 213299, China; Department of Computer Science, Northeastern University, Boston, MA 02115, USA; SecureDNA Foundation, Basel 4010, Switzerland; SecureDNA Foundation, Basel 4010, Switzerland; Institute for Interdisciplinary Information Sciences, Tsinghua University, Beijing 100084, China; Shanghai Qi Zhi Institute, Shanghai 200232, China; Department of Computer Science and Engineering, Shanghai Jiao Tong University, Shanghai 200240, China; Shanghai Qi Zhi Institute, Shanghai 200232, China; BGI Research, Changzhou 213299, China; Department of Computer Science and Engineering, Shanghai Jiao Tong University, Shanghai 200240, China

**Keywords:** DNA synthesis screening, cryptography, privacy

## Abstract

Printing custom DNA sequences is essential to scientific and biomedical research, but the technology can be used to manufacture plagues as well as cures. Just as ink printers recognize and reject attempts to counterfeit money, DNA synthesizers and assemblers should deny unauthorized requests to make viral DNA that could be misused. There are three complications. First, we do not need to quickly update printers to deal with newly discovered currencies, whereas we regularly learn of new potential pandemic viruses and other biological threats. Second, convincing counterfeit bills cannot be printed in small pieces and taped together, while preventing the distributed synthesis and subsequent re-assembly of controlled sequences will require tracking which DNA fragments have been ordered across all providers and benchtop devices while protecting legitimate customer privacy. Finally, counterfeiting can at worst undermine faith in currency, whereas unauthorized DNA synthesis could be used to deliberately cause pandemics. Here we describe SecureDNA, a free, privacy-preserving and fully automated system capable of verifiably screening all DNA synthesis orders of 30+ nucleotides against an up-to-date database of controlled sequences, and its operational performance and specificity when applied to 67 million nucleotides of DNA synthesized by providers in the USA, Europe and China.

## Teaser

To safeguard against bio-security risks posed by DNA synthesis technology, we propose a free, cryptographically secure, and fully automated system to screen all DNA synthesis requests for known potential hazards.

## INTRODUCTION

**Figure 1.**
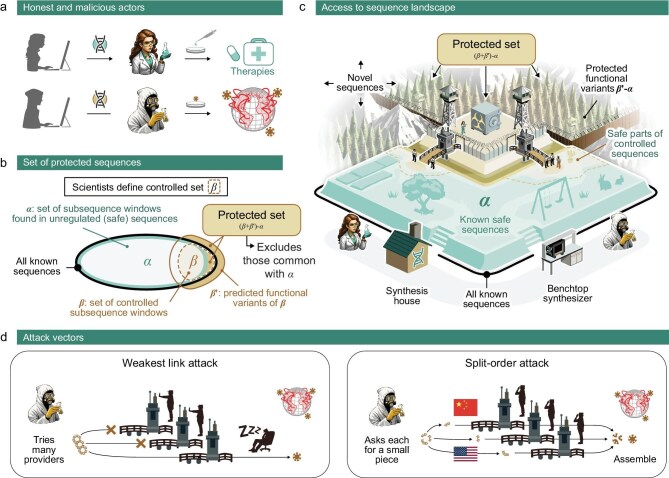
DNA synthesis screening ecosystem and security framework. (a) DNA synthesis enables therapeutic development (top), but also presents biosecurity risks (bottom). (b) DNA screening framed as a set-membership problem using configurable sets. α represents constant-length subsequences found in non-controlled (safe) sequences, including most organisms and common molecular biology tools. Scientists and regulators define the safe set α, a controlled set β, and compute functional variants β′ using any suitable variant prediction method (e.g. MCMC sampling over predicted mutation tolerance scores; see [19]). The protected set (β + β′) − α comprises subsequences from controlled sequences, or their variants, that do not appear in safe sequences. (c) Screening must cover both synthesis houses and benchtop synthesizers. Orders containing protected-set sequences require authorization. (d) A weakest link attack bypasses screening by finding a provider with inadequate security. A split-order attack evades screening by splitting controlled sequences into fragments that are either too short to screen or appear harmless in isolation. Both underscore the need for globally adopted screening of all assembly-viable sequence lengths.

Custom DNA synthesis is foundational to biomedical research, underpinning everything from cancer immunotherapies to SARS-CoV-2 vaccines. However, the same technology can also be used to produce pathogens [1–3] (Fig. 1a). The first infectious virus to be assembled from synthetic DNA was generated in 2002 at a cost of more than $10 per nucleotide [4]. Just over two decades later, the price has fallen more than a thousand-fold, the number of individuals with the necessary skills has grown from dozens to many thousands [5], and a pandemic has directly and indirectly killed over 20 million people [6], demonstrating the scale of harm that could be inflicted by a virus generated from synthetic DNA.

While no credible and accessible pandemic viruses are publicly known, numerous well-intentioned research programs aim to identify viruses capable of causing new pandemics and share their genome sequences [7–11]; one maintains a public list of viruses ranked by threat level

[12]. Future discoveries and advances in biological programming will identify other pandemic-class agents that can be generated from synthetic DNA. Because state actors can independently synthesize any DNA, the central security challenge is preventing proliferation.

The obvious response to this challenge—screening all synthetic DNA orders for a list of controlled sequences—was first recommended in 2006 [13]. Remarkably, members of the International Gene Synthesis Consortium (IGSC) voluntarily monitor an estimated 80% of global DNA synthesis, even though the relative cost of human-assisted screening is rising with volume [14,15].

Nevertheless, synthesis screening remains an unsolved global problem: (i) 36 out of 38 providers shipped DNA fragments collectively sufficient to generate the 1918 pandemic influenza virus because they had no way to know that others were supplying the remaining fragments [16]; (ii) chatbots now suggest ways to evade screening, including ordering from non-IGSC providers [17]; (iii) oligonucleotides under 50 nucleotides can be assembled and used to generate pandemic threats [14]; (iv) scientific consensus on which sequences warrant control varies across regions and evolves over time; and (v) most benchtop devices do not screen, on-device screening is insecure, and cloud-based screening [18] is not private.

The DNA industry is also at risk: (i) firms may be liable because they cannot verify that they performed best-in-practice screening; and (ii) rising demand may overwhelm expert screening with false alarms and costs may become prohibitive [14].

Solving these problems requires a free, centralized and privacy-preserving screening system configurable for any defined set of controlled sequences and effective for synthesis houses and benchtops (Fig. 1b and c).

Current screening systems rely on fuzzy-match algorithms that compare orders to both controlled and unregulated sequences, attempting to determine which they more closely resemble. This approach faces fundamental limitations; it incorrectly flags more and more unregulated sequences as the sequence window length decreases, it requires human expert review to resolve these ambiguous matches and consequently cannot be used by benchtop DNA synthesis devices, it can be evaded by introducing mutations that make controlled sequences appear more similar to unregulated genes, and it cannot reliably detect split orders distributed across providers in different nations (Fig. 1d).

Overcoming these limitations requires a new approach that can address two design challenges: (i) *bio-design*: translate the biological problem of sequence recognition into a computer science problem; and (ii) *system-design*: implement an automated system capable of verifiably screening global DNA synthesis without delaying research or compromising customer or database privacy.

In a companion paper [19], we describe a candidate solution to the first challenge: searching for exact matches to short subsequences that are unique to controlled genes and functional variants. By identifying these diagnostic fragments in advance and verifying that they do not appear in unregulated sequences, it can achieve perfect specificity among known sequences (Fig. 1b and c). This precision eliminates the need for human expert review, while the inclusion of functional variants renders screening robust against evasion attempts.

**Figure 2.**
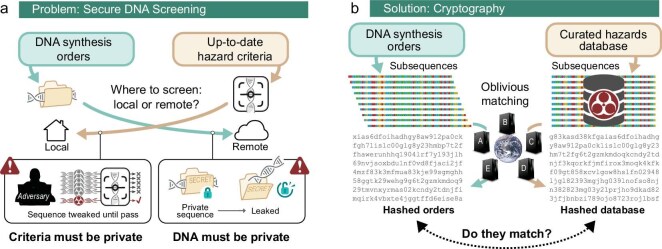
Securing DNA synthesis screening. (a) Secure and universal DNA synthesis screening requires a way to verifiably determine whether DNA synthesis orders correspond to controlled biological functions, including controlled sequences split across multiple providers and benchtop devices, without disclosing anything else about the orders. Disclosing a private customer order may compromise trade secrets, while leaking the criteria makes it possible to evade screening by introducing mutations that make a controlled sequence appear to be unregulated. These constraints cannot be satisfied simultaneously. (b) The SecureDNA system allows synthesizers and database contributors to obliviously perform one-way transformations of their subsequence windows, which can be directly compared. The database provides the synthesizer with a timestamped verification that *n* windows sent by the synthesizer were screened against a particular database version and can detect split-order attacks if adopted by most providers.

Here we address the system design challenge (Fig. 2) using a novel application of cryptography to enable centralized and privacy-preserving detection. Our automated implementation, including a graphical user interface, screens at high speed and low cost while demonstrating very high specificity, and implements a certificate system permitting authorized laboratories to seamlessly access controlled DNA.

## RESULTS

### Construction of a controlled sequence database

Scientists and regulators designate a list of DNA and peptide sequences as controlled, and construct a database of such controlled sequences. These sequences are split into overlapping windows of constant length (e.g. the first 42 characters of the text representation of a pathogen genome, then those ranging from the 2nd to the 43rd, and so on); these are defined as the controlled set β. From some of these windows known or predicted to have significant biological functions, up to 10 million variants (changes to a few characters at a time) are generated that are predicted to be at least somewhat functional, comprising the variant set β′. These are compared against a much larger set of similar constant-length sequences known to appear in nature or research in non-controlled sequences, the safe set α. The database is populated with those windows and functional variants of controlled sequences that do not appear in unregulated sequences, (β + β′) − α, or, in set notation, (β ∪ β′)∖α.

### Theoretical crypto-design and analysis

**Figure 3.**
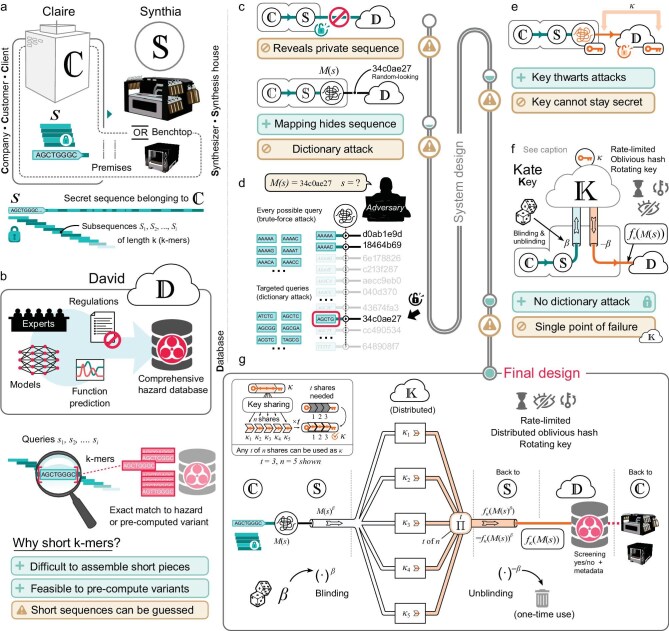
Cryptographic challenges and solutions for secure DNA synthesis screening. (a) Client Claire orders DNA from synthesizer Synthia. A sequence *s* is split into all subsequences of length *k* (*k*-mers). (b) Database David contains *k*-mers from controlled sequences and predicted functional variants. Synthia sends *k*-mers to be screened, and David detects exact *k*-mer matches. Subsequence length *k* is small to prevent assembly of unscreened fragments. (c) Synthia cannot send *s* directly to David (as in current remote DNA screening systems) without violating Claire’s privacy, but a cryptographic mapping *M*(*s*) enables secure comparison. (d) Since inputs are known to be short, a standard hash is easily cracked using a brute-force or dictionary attack. (e) A keyed hash with a secret key *κ* can thwart dictionary attacks, but requires that *κ* be known to both Synthia and David, in which case Synthia could interrogate *D* and David could crack *s*. (f) Keyserver Kate helps Synthia compute keyed hash f*_κ_*(*M*(*s*)) with oblivious cryptographic hashing. Synthia ‘blinds’ the sequence *s* with a random value β before Kate applies *κ*, and then unblinds it afterward, so no eavesdropper (including Kate) can learn *s*. Kate can limit the rate of evaluation of f*_κ_*, thwarting dictionary attacks. However, Kate is a single point of failure: an adversary compromising Kate gains *κ*, and if Kate is offline, global synthesis stops. (g) In the final design, f*_κ_*(*M*(*s*)) is distributed, making Kate’s role robust. *κ* is split into *n* key shares among *n* separated servers. Evaluating f*_κ_*(*M*(*s*)) requires a threshold number *t* of the *n* shares (*n* = 5, *t* = 3 shown). Up to (*n* − *t*) keyservers may be offline. *κ* is rotated, e.g. biweekly. Any adversary must compromise *t* servers simultaneously between key rotations to steal *κ*.

Suppose that a customer places an order for sequence *s* with a synthesizer, who wants to know whether it’s safe to make, without disclosing *s—*which may be a trade secret—to any eavesdroppers (Fig. 3a). The synthesizer can ask an up-to-date remote database *D* of controlled DNA and peptide subsequences (Fig. 3b; Appendix A in the Supplementary data) to check whether any of the constant-length DNA and translated peptide subsequences found in *s* are present in *D*. The crypto-design challenge is to find a way for the Database to answer without (i) learning anything consequential about *s* or (ii) revealing anything about *D* beyond conveying the yes/no answer, even if some of the parties among the customer and the synthesizer are compromised.

The simplest way for the synthesizer and database to privately determine whether any of their DNA or peptide subsequences are identical is to transform them using a one-way hash mapping *M* and compare the hash outputs *M*(*s*) and *M*(*D*) (Fig. 3c). But either of them could rapidly enumerate all possible short subsequences, letting them decrypt *s* and *D* (Fig. 3d), even when using a shared key *κ* (Fig. 3e). To restore privacy, we introduce the keyserver, who helps compute hashes using a frequently changed key *κ* while rate-limiting requests to match plausible DNA synthesis speeds, which renders enumeration attacks infeasible (Fig. 3f). The synthesizer and the keyserver can compute hash outputs obliviously using cryptographic techniques: the keyserver learns nothing about the input sequences or resulting hashes, while the synthesizer learns only the hash outputs.

To screen for matches, the database is given a table *H* comprising the oblivious keyed hashes of all elements in the plaintext database *D*, without learning its contents. Whenever the synthesizer submits a hashed query, the database can convey whether there are any matches to *H* without learning anything about the sequences apart from whether they match. This problem can be viewed as a special case of unbalanced private set intersection (PSI), or more precisely, a private membership testing (PMT) problem, where the querier holds a single sequence and the server holds a large set of sequences.

The database provides the synthesizer with a timestamped signature verifying that screening was performed against a specific version of *H*, allowing providers to demonstrate compliance with legal requirements and minimize liability. In addition, the database can maintain privacy-preserving logs of which controlled subsequence hashes have been detected over time, which enables detection of split-order attacks when fragments of a controlled sequence are ordered across multiple providers. Overall, this architecture ensures that no single party needs to know the list of controlled sequences or which subsequence windows are defended with functional variants—a critical feature, as knowledge of defended windows would enable evasion. In the future, the system could allow nations to screen for emerging threats without publicly acknowledging them [19].

To summarize the baseline system: (i) scientists and the keyserver build and update database *H* of obliviously hashed subsequences; (ii) the customer orders sequence *s* from the synthesizer; (iii) the synthesizer and the keyserver obliviously hash subsequences; (iv) the synthesizer sends hash outputs to the database; (v) the database tells the synthesizer if any are found in *H*; and (vi) if there are matches and the customer lacks authorization, the synthesizer refuses to make *s*.

### Security analysis of baseline screening

In the *semi-honest* model of cryptographic security, all parties follow the protocol, but may eavesdrop. Oblivious hashing ensures the keyserver learns nothing about sequences or hashes, while the synthesizer learns only whether matches exist—the minimum necessary for screening. If no controlled sequences are found, the database learns only the total sequence length (which is biologically irrelevant) and possibly whether any subsequences are shared with other clients’ queries. If *s* contains controlled sequences, the database learns which subsequences match the controlled set from *D*, but cannot determine any other sequences in *s* or *D.*

In the malicious model, a party may deviate from the protocol to eavesdrop on or sabotage its execution. A fundamental premise, however, is that the synthesizer seeks to avoid creating controlled sequences: no software-only mechanism can prevent a synthesizer that is not hardware-locked from generating controlled DNA. Commercial synthesis providers are therefore assumed to be incentivized to ensure that their internal infrastructure faithfully follows the screening workflow in order to comply with legal requirements and minimize liability. For benchtop synthesizers, stronger guarantees require hardware enforcement, such as ensuring that synthesis cannot begin until a valid screening result is returned and that the control path is protected using tamper-resistant mechanisms (e.g. techniques meeting NIST FIPS 140-2 Security Level 3 or 4 standards [20,21]). While a dishonest synthesizer could disclose the query sequence *s*, rate-limiting prevents reconstruction of the database *D* through repeated queries. A corrupt database could disclose *H* and associated statistical correlations, but cannot interrogate *s* or recover *D* without assistance from the keyserver (see Appendix B for a detailed analysis of information leakage). Conversely, a compromised keyserver could use *κ* to hash all possible subsequences and thereby recover information about *s* and *D*.

### Distributing the key

The integrity of the keyserver is so vital that we divide the key *κ* into *n* shares and distribute them across *n* keyservers using Shamir secret sharing [22] to avoid a single point of failure (Fig. 3g). This architecture requires a threshold of *t* keyservers to jointly perform oblivious hashing: any subset of *t* keyservers can jointly compute the keyed hash with the synthesizer Synthia, but any smaller coalition learns nothing about *κ*. The SecureDNA PMT protocol’s main efficiency gain over traditional PSI comes from introducing this set of trusted third parties who pre-process parts of the computation offline. To prevent a malicious party from stealing *κ* by gradually corrupting the majority of the keyservers, the system periodically updates key shares through a coordinated process with the same security guarantees as distributed oblivious hashing—no single party ever reconstructs *κ.* This achieves what cryptographers call *proactive security; κ*, and thereby all *s* and *D*, remain protected even if all keyservers are eventually hacked, as long as they are not compromised simultaneously. See Appendix C for the complete protocol.

### System design and implementation

The database is constructed by partitioning controlled sequences into 30 or 42 nucleotide DNA windows and 20 amino acid peptide windows (β), which are tagged with geographical regions in which they are regulated. For a subset of windows, chosen pseudo-randomly, we stochastically include mutants and functional variants predicted using protein structure and sequence homology (β′).

To ensure specificity among all known sequences, we check each candidate subsequence against a dataset of nucleotides and proteins derived from NCBI’s nr/nt GenBank and GenPept databases and remove any that are also found in an unregulated sequence (i.e. remove any in α). This filtering process combines taxonomic analysis, keyword identification (e.g. ‘synthetic’ or ‘recombinant’) and controlled sequence coverage metrics to improve database annotations. An analysis of controlled viral sequences revealed that nearly all contained thousands of *k*-mers (*k* ≥ 30) unique to strains of the controlled virus, providing ample targets for effective screening.

The system can accommodate any arbitrarily defined set of controlled sequences, permitting customization by provider preference. By default, it detects controlled sequences defined in Australia Group, International Traffic in Arms Regulations (ITAR) or national regulations, viruses described as potential pandemic pathogens in the scientific literature, and viruses capable of human-to-human transmission or illness that could be misused. While the system returns an alert upon any unique match, it recommends denying synthesis for controlled viruses, potential pandemic viruses, genes encoding controlled toxins, and sequences that could confer controlled-pathogen capabilities to more accessible strains per the Australia Group’s ‘endow or enhance pathogenicity’ definition.

### Split-order detection

Even modestly resourced actors can evade screening by splitting controlled sequences into multiple individually harmless fragments and ordering them from different providers, who have no way of knowing whether others are making the remaining pieces [16]. SecureDNA’s primary defense against such split-order attacks is fragment-level screening at short sequence lengths, which substantially constrains assembly-viable evasion strategies. However, detecting accumulation of fragments associated with a particular controlled sequence across globally distributed providers remains important for identifying coordinated misuse attempts. Even if governments required logs of all controlled fragments ordered from their providers, nations would need to share data with one another to detect such attacks, which appears unlikely given diplomatic constraints. Because it is privacy-preserving, offered free of charge by a non-profit organization in a neutral country, co-developed internationally, and can be used by centralized synthesis houses and benchtop synthesizers, SecureDNA could potentially be adopted widely enough to detect such attempts by monitoring which pieces have been ordered from different suppliers and notifying either suppliers or law enforcement of detected attacks, as appropriate. Providers can then strictly require evidence of authorization for any requests for the remaining pieces, including pausing any in-progress orders, thereby denying access to the adversaries attempting the split-order attack. To enable split-order detection, the system stores hashes of controlled subsequences in order and logs which ones have been detected, while learning nothing of which customer ordered which *k*-mers, only which provider they ordered it from. Statistical accumulation patterns over time can then be used to support escalation or trigger additional scrutiny, without requiring customer identity matching.

### System operation

**Figure 4.**
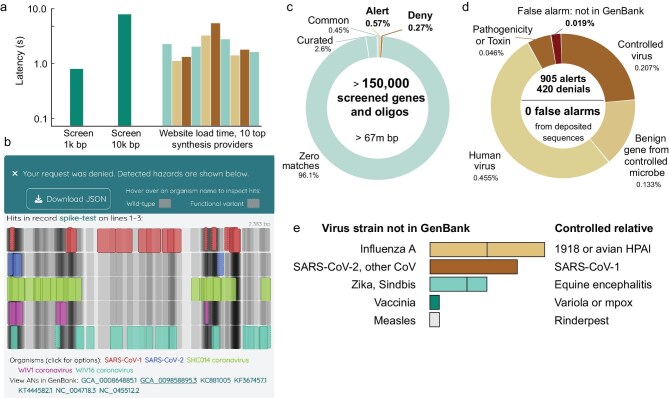
SecureDNA latency, output and specificity of real-world synthetic genes. (a) A comparison of the time required to screen 1000- and 10 000-bp orders to the typical provider website response times. SecureDNA does not meaningfully delay order placement and can return results to customers in real time. (b) Output showing matches to wild-type and variant windows from controlled pathogens with hover-over interface for inspection, including GenBank references and flagged sequences from human viruses and benign genes from regulated bacteria. The depicted chimera was denied because it includes *k*-mers unique to SARS-CoV-1. (c) Analyzing >67 million nucleotides of synthesized genes from multiple providers with SecureDNA flagged 0.57% of genes (yellow) and denied 0.27% (brown). Fragments present in both controlled (β or β′) and safe (α) sequences are curated, while sequences unique to β but often used in biotechnology (e.g. P2A peptides) are common. Both represent matches but not alerts. (d) Iterative manual BLAST revealed zero false alarms from known sequences: all 30 false alarms were from strains of related viruses not present in GenBank. (e) Probable identities of the 30 false alarms (1 in 5000 orders) flagged due to sequence windows not present in GenBank.

Each synthesizer or provider running the open-source SecureDNA *synthclient* program converts FASTA files from sequence orders into all eligible subsequences and translations (assuming the canonical genetic code; alternative genetic codes do not enable access to transmissible pandemic agents). Screening proceeds in both forward and reverse-complement directions across the query sequence [19] and, for benchtop synthesizers, includes base permutations to prevent evasion through reagent bottle swapping (Fig. S1). This step negligibly reduces specificity and actually improves performance, as all 48 possible hash combinations (including all possible base permutations in both directions) are consolidated. After local processing, *synthclient* contacts the keyservers to perform oblivious hashing and sends the results to the database for comparison. Screening completes faster than most webpages load (Fig. 4a), providing an immediate ‘accepted’, ‘alert’ or ‘denied’ decision along with a signed, timestamped verification that screening was performed using a specific database version. The system also generates a visual mapping of any matches to public controlled sequences and notes relevant export control restrictions (Fig. 4b).

### Deployment and performance

The production system operates with five keyservers distributed across multiple geographic zones to maximize fault tolerance, requiring any three to process requests. Under normal load, the system completes key rotation every 1–2 weeks, with the capacity to recompute the entire database *H* in 3 days if needed. The primary computational bottleneck remains elliptic curve group operations, requiring 10–1000 μs per window depending on architecture.

Despite modest computational resource requirements (around $5000–$8000 per year; see Methods), the system demonstrates robust performance. *Synthclient* processes 1100 nucleotides per second (nt/s) per CPU thread, or about 2200 nt/s per hyperthreaded core, on provider hardware. Keyservers achieve 4400 nt/s on 4-thread CPUs. The database, using cloud SSD storage, handles 41 500 nt/s per disk, and over 74 000 nt/s in a physical machine with NVMe storage, with constant-time lookup regardless of database size up to 15× the size of the current database. Most providers need only a single $2000 desktop computer or can screen for pennies per day using on-demand cloud servers, while benchtop synthesizers can operate with a $50 Raspberry Pi 4B (Table S1). For customers with very large oligo-manufacturing needs, we have demonstrated 40 million nucleotides in under 10 min at a customer cost of 61 US cents for cloud-based clients. The current hardware configuration can screen more than 4 trillion nucleotides annually—sufficient for projected global synthesis volume through 2029. As SecureDNA expands, we plan to increase security and redundancy by adding servers (Appendix D). Operating fees estimated at $15 000–$30 000 per year (depending on degree of redundancy) for servers housed in a colocation facility constitute the only recurring usage cost borne by SecureDNA (Appendix D), and are sufficient to support an order of magnitude more screening than the estimated size of the current market for both genes and oligos.

### Quantifying specificity using real-world synthesis data

To measure specificity in a real-world context, we screened anonymized orders for over 150 000 genes and oligonucleotides synthesized by providers in the USA, Europe and China, which together comprise over 61 million nucleotides generated for biological research and 6 million for DNA storage.

Over 99% of sequences passed screening, and the overwhelming majority (96%) featured zero matches. Among sequences with one or more matches (4%), two-thirds (2.6%) were found in unregulated sequences and were not flagged (Fig. 4c). One-third of those remaining (0.45%) were flagged as ‘common’ because they matched one of the many frequently used sequences in biotechnology that originated in a regulated organism or a human-infecting virus. Examples include the porcine teschovirus 2A peptide and the cytomegalovirus enhancer/promoter.

SecureDNA identified genes with at least one nucleotide or peptide match to a controlled organism or human virus in 0.85% of sequences (Fig. 4c). Each was investigated by running BLAST on the entire gene sequence, then on any subsequences that were not clearly identified in the first attempt, and manually labeling (Fig. 4d). Approximately half (0.455%) were from unregulated viruses capable of infecting humans, for which several providers have requested alerts; others (0.133%) matched benign genetic sequences from regulated microbes that could not confer pathogenicity upon more easily obtained related or avirulent strains. With respect to the controlled sequences included in the evaluation corpus (e.g. U.S. Select Agents and Australia Group pathogens), we observed zero false negatives.

In the absence of an exemption token (ET; see below), we would have recommended denying just 0.272% of all ordered genes; 0.046% encoded a toxin or conferred pathogenicity upon a more easily obtained avirulent microbe, 0.207% were unique to a controlled or potential pandemic virus, and 0.019% (30 gene fragments) were from strains of unregulated human-infecting viruses not deposited in GenBank (Fig. 4d), all related to controlled viruses.

Crucially, we observed zero false alarms from known sequences in GenBank; the system perfectly distinguished between all known sequences. The 30 false alarms from sequences absent from GenBank were all from strains of related human-infecting viruses or deliberately created mutants that had never been deposited. We estimate that at current DNA synthesis rates, this false alarm rate would correspond to just ∼4 wrongly denied sequences globally each day. By comparison, we anticipate approximately 50 legitimate authorization requests daily for controlled sequences, meaning more than 90% of all authorization requests would be for actual controlled sequences rather than false alarms. Nevertheless, it is imperative for authorized laboratories that work with these pathogens—or with controlled agents—to gain access to the DNA that they need to advance human knowledge and develop therapies, without delay.

### Automating customer screening and permissions

Members of the IGSC spend considerable time and effort screening their customers for legitimacy. In some cases, providers contact the customer’s local biosafety authority to ask whether the customer is authorized to work with a particular controlled organism.

**Figure 5.**
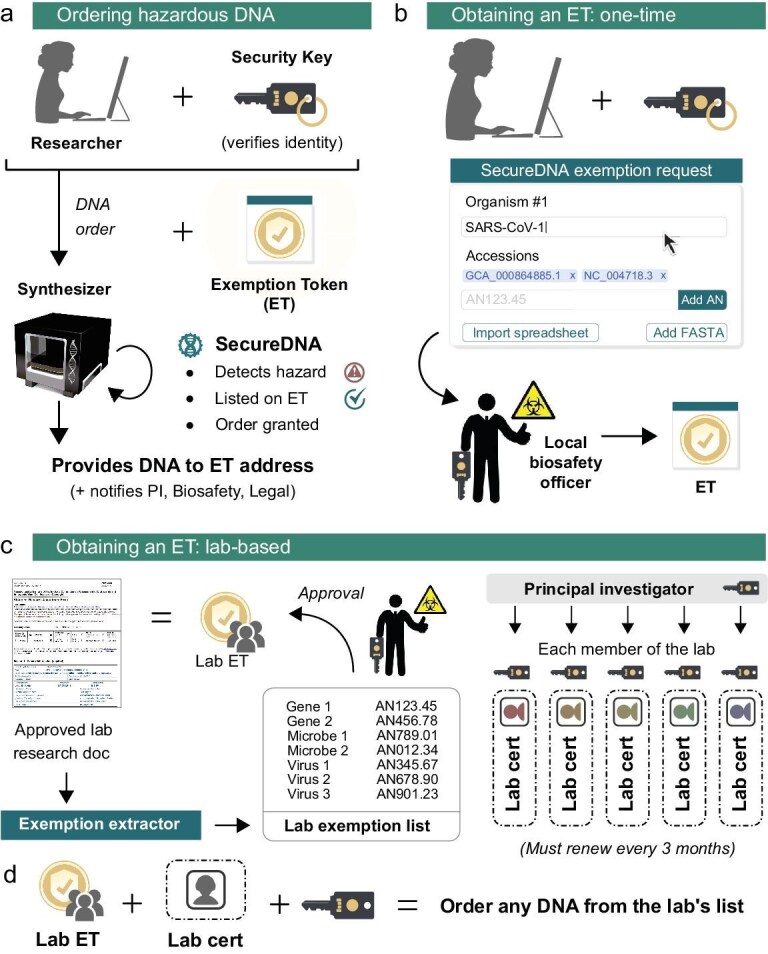
SecureDNA exemption lists for automated customer screening. (a) Researchers with a hardware security key can submit an ET with their order to bypass denials for any controlled sequence listed on the ET. (b) To obtain a one-time ET, researchers can specify a controlled agent from a drop-down table, enter a GenBank accession number, or import a DNA sequence, and then send it to their local biosafety officer to be approved and signed using the institution’s certificate. (c) Labs can obtain an ET that will work for all members by using an extraction tool on their research registration document to obtain a list of genes, microbes and viruses listed in the document, which can then be approved by the biosafety officer. The lab principal investigator can grant ‘lab certificates’ to each member. (d) A lab ET, lab certificate and matching security key can obtain DNA from any gene or organism the lab is approved to work with, including not-yet-deposited strains and predicted functional variants.

An ideal screening system would automatically grant researchers access to DNA corresponding to pathogens that their biosafety authority has already approved them to work with. SecureDNA allows researchers to submit an ‘exemption token’ (ET) with their order to obtain any permitted controlled DNA (Fig. 5a). A one-time token contains a public key identification (PKI) certificate instructing the database to ignore matches corresponding to exempted controlled sequences, but only for requests originating from the user. This allows researchers to swiftly obtain DNA corresponding to any controlled gene, organism or set of raw sequences by requesting an exemption from their biosafety officer, who approves it using their own security key and a certificate issued by a higher biosafety authority, such as one at the national level (Fig. 5b, Fig. S2).

To facilitate seamless ordering, we wrote software to help biosafety authorities convert approved laboratory research registration documents into ETs for permitted genes and organisms, then issue the lab with an ET (Fig. 5c). The principal investigator can separately grant certificates to lab members, enabling them to automatically bypass SecureDNA denials of sequences on the lab exemption list as long as they are shipped to the lab’s address (Fig. 5d). In all cases, a physical hardware authentication key ($20–$30) or time-based one-time password (TOTP) from an authentication app is required to validate the user’s identity, and placing an order for an exempted controlled sequence notifies the principal investigator, the biosafety officer and the organization’s legal contact.

For example, suppose that a laboratory wants to develop a broad-spectrum influenza vaccine and needs access to sequences from 1918 influenza. Using the exemption request tool, they can select ‘1918 influenza’, then send the request to their biosafety authority (Fig. S3). Once approved, every lab member can use the ET and their personal certificate proving lab affiliation to ship any DNA sequences corresponding to that strain to the lab’s registered address, or synthesize them on a benchtop device. Researchers can also request exemptions covering lists of sequences, such as oligonucleotide libraries for deep mutational scanning [23] and directed evolution [24]. Importantly, the database tracks which pieces of controlled sequences have been synthesized by any provider, with or without exemptions, in order to detect split-order attacks.

As a final precaution, whenever an order for a public controlled sequence is automatically approved using an exemption list, the SecureDNA database notifies the lab’s principal investigator, biosafety authority and institutional legal department for record-keeping purposes. Because biosafety vetting and approval are available as a commercial service [25], researchers anywhere in the world can access the benefits of seamless and secure ordering via automated customer screening.

## DISCUSSION

Progress in the life sciences is demonstrably vulnerable to public mistrust [26]. A pandemic deliberately caused by a malicious actor would almost certainly trigger a backlash and draconian research restrictions. Safeguarding the promise of biotechnology requires a way to ensure controlled DNA is only shipped to legitimate laboratories [27,28], but the complexity of the problem and the expense of regulation have deterred international action.

The advent of free, automated, benchtop-compatible and privacy-preserving DNA synthesis screening (Table S2) may allow nations currently hesitant to place their own companies at a competitive disadvantage to begin regulating the sector and clarifying liability in the event of misuse. Indeed, signatories of the Biological Weapons Convention may be obligated to require free screening under Article IV, which states that parties must ‘take any necessary measures to prohibit and prevent the development, production, stockpiling, acquisition, or retention of the agents, toxins, weapons, equipment and means of delivery… within the territory of such State, under its jurisdiction or under its control anywhere’.

While DNA synthesis screening may prevent widespread access to credible pandemic agents for many years, advances in *de novo* protein design [29–31] will gradually undermine its effectiveness. Design models can already generate functional equivalents of binding proteins; while this presumably includes toxins, they are far less relevant to international security than pandemic agents. However, *de novo* biodesign tools may eventually be capable of generating allosteric or catalytic proteins sufficient to enhance natural pathogens or even produce novel pandemic agents. Requiring that requested novel biomolecules be demonstrably consistent with a declared research intent could offer limited protection, but such intent-based checks are easily circumvented by benign explanations and do not scale to complex or exploratory research. Moreover, function and folding prediction tools that rely on a complete and intact sequence will not be able to detect such threats if the resulting synthetic genes are ordered in pieces, among other evasive strategies (Fig. S4). Finally, denying all unknown DNA as a blanket strategy would preclude routine use of optimized linkers, spacers, randomized regions and other legitimate novel sequences, effectively banning a wide range of legitimate research.

Controlling access to protein and genome design tools via application programming interfaces (APIs) and logging controlled designs for screening purposes, which could be done for nearly all users even if source code was shared among all registered developers, may help prevent redesigned versions of controlled agents from evading detection. To further mitigate security risks, leaders in the protein design community have called for the retention of cryptographic records of DNA synthesis orders [32], which could deter malicious actors by reliably identifying the source of the harmful DNA after the fact. Systems running SecureDNA could readily store order hash records, with old keys divided among trusted authorities after rotations and released only following a catastrophic event, to enable forensic screening of past synthesis activity while maintaining day-to-day privacy [32].

Collectively, our results suggest that SecureDNA can provide free, private, reliable and verifiably up-to-date nucleic acid synthesis screening at a scale sufficient to meet global demand, with negligible false alarms and a largely automated authorization process. If supported by favorable regulatory and liability policies, hardware integration into next-generation synthesizers, and subsidized trade-in programs, near-universal screening could dramatically reduce unauthorized access to pandemic-class agents without delaying research. By preventing the conversion of controlled blueprints into dangerous pathogens, we can safeguard biotechnology and the world from the threat of deliberate pandemics.

## Supplementary Material

nwag103_Supplemental_File

## Data Availability

The open-source SecureDNA software is available at https://github.com/SecureDNA/. The privacy of customer data from historical DNA synthesis orders is protected by legal agreements signed by the relevant providers. We are not aware of any open data sources, and encourage interested parties to reach out to providers directly to discuss the possibility of signing non-disclosure agreements.

## Notes

Authors are listed in alphabetical order by surname.

## References

[1] Neumann G, Ozawa M, Kawaoka Y. Reverse genetics of influenza viruses. Methods Mol Biol 2012; 865: 193–206. 10.1007/978-1-61779-621-0_12 22528161

[2] Maroun J, Muñoz-Alía M, Ammayappan A et al. Designing and building oncolytic viruses. Future Virol 2017; 12: 193–213. 10.2217/fvl-2016-0129 29387140 PMC5779534

[3] Xie X, Lokugamage KG, Zhang X et al. Engineering SARS-CoV-2 using a reverse genetic system. Nat Protoc 2021; 16: 1761–84. 10.1038/s41596-021-00491-8 33514944 PMC8168523

[4] Cello J, Paul AV, Wimmer E. Chemical synthesis of poliovirus cDNA: generation of infectious virus in the absence of natural template. Science 2002; 297: 1016–8. 10.1126/science.1072266 12114528

[5] Geneva Centre for Security Policy, Esvelt KM. Delay, detect, defend: preparing for a future in which thousands can release new pandemics. https://www.gcsp.ch/publications/delay-detect-defend-preparing-future-which-thousands-can-release-new-pandemics (20 January 2026, date last accessed).

[6] Mathieu E, Ritchie H, Rodés-Guirao L et al. COVID-19 Pandemic. https://ourworldindata.org/coronavirus (20 January 2026, date last accessed).

[7] Carroll D, Daszak P, Wolfe ND et al. The Global Virome Project. Science 2018; 359: 872–4. 10.1126/science.aap7463 29472471

[8] Warren CJ, Yu S, Peters DK et al. Primate hemorrhagic fever-causing arteriviruses are poised for spillover to humans. Cell 2022; 185: 3980–91. 10.1016/j.cell.2022.09.022 36182704 PMC9588614

[9] Sun H, Li H, Tong Q et al. Airborne transmission of human-isolated avian H3N8 influenza virus between ferrets. Cell 2023; 186: 4074–84. 10.1016/j.cell.2023.08.011 37669665

[10] Hou YJ, Chiba S, Leist SR et al. Host range, transmissibility and antigenicity of a pangolin coronavirus. Nat Microbiol 2023; 8: 1820–33. 10.1038/s41564-023-01476-x 37749254 PMC10522490

[11] Thadani NN, Gurev S, Notin P et al. Learning from prepandemic data to forecast viral escape. Nature 2023; 622: 818–25. 10.1038/s41586-023-06617-0 37821700 PMC10599991

[12] Grange ZL, Goldstein T, Johnson CK et al. Ranking the risk of animal-to-human spillover for newly discovered viruses. Proc Natl Acad Sci USA 2021; 118: e2002324118. 10.1073/pnas.2002324118 33822740 PMC8053939

[13] Bügl H, Danner JP, Molinari RJ et al. DNA synthesis and biological security. Nat Biotechnol 2007; 25: 627–9. 10.1038/nbt0607-627 17557094

[14] Diggans J, Leproust E. Next steps for access to safe, secure DNA synthesis. Front Bioeng Biotechnol 2019; 7: 86. 10.3389/fbioe.2019.00086 31069221 PMC6491669

[15] Beal J, Clore A, Manthey J. Studying pathogens degrades BLAST-based pathogen identification. Sci Rep 2023; 13: 5390. 10.1038/s41598-023-32481-z 37012314 PMC10068195

[16] Edison R, Toner S, Esvelt KM. Evaluating the adequacy of DNA synthesis screening. https://drive.google.com/file/d/1hNUnU8i2yubt5uesmmV17aTJXhYYDgTY/view?usp=drive_link (20 January 2026, date last accessed).

[17] Soice EH, Rocha R, Cordova K et al. Can large language models democratize access to dual-use biotechnology? [preprint] arXiv: 2306.03809.

[18] Nouri A, Chyba CF. DNA synthesis security. Methods Mol Biol 2012; 852: 285–96. 10.1007/978-1-61779-564-0_21 22328441

[19] Gretton D, Wang B, Edison R et al. Exact-match search with functional variant prediction enables automated DNA screening [preprint]. bioRxiv: 2024.03.20.585782.

[20] National Institute of Standards and Technology . FIPS PUB 140-2. Security Requirements for Cryptographic Modules. https://nvlpubs.nist.gov/nistpubs/FIPS/NIST.FIPS.140-2.pdf (20 January 2026, date last accessed).

[21] National Institute of Standards and Technology . Implementation Guidance for FIPS 140-2 and the Cryptographic Module Validation Program. https://csrc.nist.gov/csrc/media/projects/cryptographic-module-validation-program/documents/fips140-2/fips1402ig.pdf (20 January 2026, date last accessed).

[22] Shamir A . How to share a secret. Commun ACM 1979; 22: 612–3. 10.1145/359168.359176

[23] Fowler DM, Fields S. Deep mutational scanning: a new style of protein science. Nat Methods 2014; 11: 801–7. 10.1038/nmeth.3027 25075907 PMC4410700

[24] Packer MS, Liu DR. Methods for the directed evolution of proteins. Nat Rev Genet 2015; 16: 379–94. 10.1038/nrg3927 26055155

[25] ABSA International. Biosafety Buyer’s Guide—Biosafety Consultants. https://biosafetybuyersguide.org/consultants.html (20 January 2026, date last accessed).

[26] Lewis T . The quest to overcome gene therapy’s failures. Nature 2021; 10.1038/d41586-021-02734-w (20 January 2026, date last accessed). 10.1038/d41586-021-02734-w 34703014

[27] Carter S, DiEuliis D. Mapping the Synthetic biology industry: implications for biosecurity. Health Secur 2019; 17: 403–6. 10.1089/hs.2019.0078 31593512

[28] DiEuliis D . Revisiting the Digital Biosecurity Landscape. In: Greenbaum D (ed.). Cyberbiosecurity. Cham: Springer, 2023, 71–8. 10.1007/978-3-031-26034-6

[29] Watson JL, Juergens D, Bennett NR et al. *De novo* design of protein structure and function with RFdiffusion. Nature 2023; 620: 1089–100. 10.1038/s41586-023-06415-8 37433327 PMC10468394

[30] Alamdari S, Thakkar N, Berg R et al. Protein generation with evolutionary diffusion: sequence is all you need [preprint]. bioRxiv: 2023.09.11.556673.

[31] Chen B, Cheng X, Li P et al. xTrimoPGLM: unified 100B-scale pre-trained transformer for deciphering the language of protein [preprint]. arXiv: 2401.06199.

[32] Baker D, Church G. Protein design meets biosecurity. Science 2024; 383: 349. 10.1126/science.ado1671 38271530

